# Increasing the Usability of the American Time Use Survey: IPUMS ATUS

**DOI:** 10.3390/data10110189

**Published:** 2025-11-14

**Authors:** Kari C. W. Williams, Sarah M. Flood, Liana C. Sayer, Julia A. Rivera Drew

**Affiliations:** 1 IPUMS Center for Data Integration, University of Minnesota, Minneapolis, MN 55455, USA; 2 Maryland Time Use Laboratory, University of Maryland, College Park, MD 20742, USA

**Keywords:** time use, IPUMS, harmonization, data infrastructure, data access

## Abstract

This paper describes IPUMS ATUS, which simplifies the use of time diary data by disseminating a harmonized and enhanced version of the American Time Use Survey (ATUS). The ATUS time diary data capture the detailed activities over a 24 h period for thousands of respondents along with their sociodemographic characteristics. The ability to measure, at a population level, how people spend their time provides nearly endless possibilities for examining questions that hinge on understanding human behavior. The flexible data format can be used to estimate time use as captured by stylized survey questions (e.g., sleep duration, work hours), but it also allows for the study of activity sequencing and the context of time use (e.g., where it happens, who else is present). However, wrangling the complex, hierarchical record structure of the data requires advanced programming skills. To address these challenges, IPUMS ATUS harmonizes the ATUS data and provides customization tools that allow researchers to (i) combine data from multiple original ATUS files and (ii) easily create and save custom variables that summarize time use utilizing the full array of contextual information spread across the complex record structure of the ATUS.

## 1. Summary

Time diary data capture temporal and contextual details about time use in people’s daily lives using a structured format that allows for quantitative analysis of daily life at scale. The American Time Use Survey (ATUS), released annually since 2003, enables detailed examinations of how Americans spend their time. This rich data source provides incredible flexibility to estimate time use as captured by stylized survey questions (e.g., sleep duration, work hours), but also allows for the study of activity sequencing and timing as well as the context of time use (e.g., where it happens, who else is present). The richness of the data comes at a cost: a complex, hierarchical record structure. IPUMS, which harmonizes census and survey data from the United States and around the world, disseminates a harmonized version of the ATUS and provides tools that increase the accessibility of these valuable data. In this paper, we will provide an overview of time diary data from the ATUS, then describe the IPUMS ATUS harmonization and software that unlock the full potential of the ATUS data. We will then highlight the range of potential applications of IPUMS ATUS data, key considerations for data users, and upcoming work that we plan to further the use of these valuable data.

## 2. Data Description

### 2.1. ATUS Time Diary Data

The ATUS is the first ongoing, federally administered survey on time use in the United States. The survey is sponsored by the United States (U.S.) Bureau of Labor Statistics (BLS) and fielded by the U.S. Census Bureau. Data have been collected and released annually since 2003. The ATUS sample is drawn from the households that have completed the eighth interview of the Current Population Survey (CPS) panel. For each household sampled by the ATUS, one person aged 15 or older is selected to be interviewed one time. Respondents report how they spent their time on the previous day, where they were, and who they were with. In addition to these questions about time use, the survey gathers information about the respondent and their household members at the time of the ATUS interview. The ATUS data files also include demographic information from the household’s final CPS interview. Beginning in 2011, the survey began collecting data on adult caregiving through questions specifically designed to identify persons who provide eldercare. A number of rotating modules capture additional information on eating and health, well-being, as well as work schedules and paid/unpaid leave. The ATUS is fielded continuously, with approximately 10,000 respondents participating annually and more than 244,000 respondents from 2003 to 2024.

The ATUS is unique among time diary data sources in the U.S. and around the world. It is the only comprehensive source of data on how people in the U.S. spend their time. The data are population representative, and the ATUS boasts both large sample sizes and two decades of consistent coverage. Other U.S. time diary data collection efforts, both historical and contemporary, are smaller, stand-alone datasets that may focus on a particular population (e.g., parents, cohabitors) rather than a comprehensive series intended to represent the U.S. population. The ATUS is the only nationally representative time diary data collected on an annual basis in the world, and data collection for the survey is continuous throughout each year. Among countries that collect time diary data, the data are generally fielded at regular, but not annual, intervals and many do not gather data over the full course of the year.

The IPUMS ATUS version of the ATUS data broadens access and reduces barriers to using these rich yet complex data. We describe the benefits of IPUMS ATUS—harmonization, data access system, custom time use variables, linking to CPS, and reproducibility—in greater detail under Section 3.

#### 2.1.1. Sample Design

The ATUS is representative of the resident, non-institutionalized, civilian population of the U.S. Drawn from the households that are completing their participation in the CPS, the ATUS sample is selected in three steps; each of these is described in greater detail in the American Time Use Survey’s User Guide [1]. First, some of the sample in less populous states that are oversampled by the CPS, which, unlike ATUS, is required to be representative at the state level, is removed while ensuring the remaining sample is representative of the U.S. The remaining sample is then stratified by race/ethnicity of the householder, presence and age of children, and the number of adults in adult-only households. Households with a Hispanic or non-Hispanic Black householder and households with children are oversampled to improve estimates for these groups. For each household in the ATUS, one randomly selected person aged 15 or older is asked to complete a 24 h time diary. Individuals are assigned a diary day, with 10% allocated to each of the weekdays (Monday through Friday), and 25% of diary days allocated to Saturday and Sunday, respectively. Allocating diary days in this way reflects research demonstrating that time allocation is similar across weekdays, but differs both between weekdays and weekends and between weekend days, and ensures adequate numbers of weekend days for generating reliable estimates [2].

#### 2.1.2. Data Collection

ATUS interviews are conducted two to five months after the ATUS household completes their final CPS interview. Interviews are administered by telephone in English or Spanish. Selected respondents who cannot be reached on their assigned interview date are re-contacted; they are called the same day the following week for up to eight weeks. The telephone interviewer leads the respondent through their activities over the 24-h period beginning at 4:00 a.m. on the designated diary day through 3:59 a.m. the following day. Respondents describe their primary activity in their own words, listing their activities sequentially and reporting an ending time or length for each activity. Interviewers record the verbatim responses, which are then coded into an activity classification system representing more than 400 detailed activities [3]. For example, “watching basketball” is assigned a different code than “playing basketball.” In contrast, both “sleeping” and “napping” are coded as “sleeping” in the ATUS and assigned the same code. Additional details about the activity context, including where activities occurred, who else was present, and whether select secondary activities (like eldercare) were being performed at the same time, are also collected during the interview. To be considered a valid response, respondents must report at least five activities; the average number of activities reported by ATUS respondents is 19.

Given the interest of BLS in paid and unpaid work, other summary questions are used to identify activities that are related to work and volunteering through an organization. Specifically, respondents are prompted to identify activities performed as part of their job or for generating income, and to identify activities performed for or through an organization. Beginning in 2011, ATUS respondents are also asked whether they provided any care or assistance in the previous three months for an adult who “needed help because of a condition related to aging” [4] (p. 33). The questionnaire does not specify a minimum age for these eldercare recipients, but it does describe what types of activities might be considered eldercare: “For example, as people grow older, it sometimes becomes difficult for them to perform various activities without help — such as grooming, driving, managing the household, taking medication or other common activities” [4] (p. 33). Respondents who reported that they had provided eldercare then completed a series of questions about the number of people for whom they provide care, the time spent in eldercare, basic information about the eldercare recipient, and how long the respondent has provided care.

The ATUS occasionally includes modules following the completion of the main interview. Three topical modules have been fielded multiple times each since the ATUS began. Module non-response is typically quite low. The Eating and Health and Well-Being modules are designed for all respondents while the Leave and Job Flexibilities module is limited to employed wage and salary workers. With the exception of the 2021 Well-Being module, the data collection spans the entire calendar year in line with the main ATUS.

**The Eating and Health module**, collected in 2006–2008, 2014–2016, and 2022–2023, is sponsored by the Economic Research Service, which is part of the U.S. Department of Agriculture. The module collects data on secondary eating and other topics to enable analyses of time use alongside obesity, participation in food and nutrition assistance programs, grocery shopping, and meal preparation.

**The Leave and Job Flexibilities module**, collected in 2011, 2017–2018, and 2024, is sponsored by the U.S. Department of Labor Women’s Bureau. The module focuses on employed wage and salary workers and assesses their access to and use of paid and unpaid leave, job flexibility, and work schedules and locations.

**The Well-Being module** was fielded in 2010, 2012, 2013, and 2021. The 2010–2013 data collections were sponsored by the National Institute on Aging, and the 2021 data collection was sponsored by the University of Minnesota and the University of Maryland with support from the National Science Foundation and the Eunice Kennedy Shriver National Institute of Child Health and Human Development. The 2010–2013 modules were fielded the entire year while the 2021 data collection spanned the 1 March 2021 to 31 December 2021 period. The module is focused on quality of life, including life satisfaction, health, and well-being. These data capture well-being during three randomly selected activities (excluding sleeping, grooming, and personal activities) from the time diary. Respondents report their levels of happiness, fatigue, sadness, stress, pain, and meaning for each activity.

In addition to collecting information from the ATUS respondents about their time use and their latest demographic and labor force characteristics, ATUS respondents also specify who they live with (i.e., a household roster) and their non-resident children. Respondents then provide very basic information about other household members, such as their age, sex, and relationship to the respondent. Additional information about most ATUS respondent household members is available from the CPS. A subset of these demographic variables collected during the final CPS interview are delivered directly with the ATUS data files. All CPS data are available through linkage (see Linking to CPS section), which requires additional data management and knowledge of an additional complex data collection.

#### 2.1.3. Data Format

The core of the ATUS—and what makes it distinct from other federal data collections and other types of survey-based data collection—is the time diary component. Time diary data are an appropriate, if not potentially superior, substitute for stylized time use questions in other surveys and offer tremendous detail and flexibility not available in other data sources [5]. The time diary contains the details about what respondents did and when, where they were, who they were with, and any additional information on secondary activities. The richness of these data necessitates a complex, hierarchical relationship between different elements of the data file. The original data provided by the BLS, which are available at https://www.bls.gov/tus/data/datafiles-2024.htm (accessed on 15 September 2025), stores, for example, the 2024 ATUS data in seven basic data files and three additional data files. For delivery via IPUMS and parsimony, IPUMS ATUS organizes the multiple original data files into a single, multilevel hierarchical file. Table 1 provides a visual description of the five types of records in IPUMS ATUS, how the record types nest within one another, and their relationship to the data files available from BLS^1^. Note that the “Respondent” and “ATUS-CPS” files both contain data about households and persons. Five original files—“Respondent,” “Roster,” “ATUS-CPS,” “Case History,” and “Weights”—each contain data at the level of individuals.

**Activity Records** provide the building blocks of the time diary data. Respondents’ own descriptions of their activities are classified into a detailed activity coding scheme with more than 400 coded activities that facilitates grouping of broad categories of activities and disaggregation within each category [4,6]. The full ATUS activity coding structure has three tiers. The top-tier consists of 17 two-digit major activities: personal care; household activities; caring for and helping household members; caring for and helping non-household members; working and work-related activities; education; consumer purchases; professional and personal care services; household services; government services and civic obligations; eating and drinking; socializing, relaxing, and leisure; sports, exercise, and recreation; religious and spiritual activities; volunteer activities; telephone calls; and traveling.

Each of these major activities is further disaggregated with two-digit intermediate codes and two-digit detailed activity groupings. Consider, for example, household activities (see Table 2) which are represented by major activity code “02”. Household activities are disaggregated into ten intermediate categories (e.g., housework, vehicles) which contain even more detailed activity codes (e.g., housework includes cleaning and laundry among other activities). For each activity, the data also include the start and end times, duration in minutes, location, who else was present, and whether or not the respondent was engaged in a secondary activity.

For each primary activity reported, respondents are also asked to report additional information about the context of the activity. For most activities—excluding sleeping, grooming, and some personal activities—respondents are asked to provide a location or mode of transportation. We present a truncated sample time diary in Table 3. It includes the data as formatted by IPUMS ATUS (with the exception of the Who records as described in the footnote of Table 3) and demonstrates the contextual detail available for a person’s activities.

**Secondary Activities** are those that are undertaken simultaneously with the primary activity. They are generally not recorded in the ATUS. Exceptions include secondary childcare, which is available in all years, and secondary eating, which is part of the Eating and Health module [7]. Secondary childcare and secondary eating data are gathered through summary questions at the end of the survey that ask about all periods of the diary day when the respondent had a child under the age of 13 in their care or was eating while they were doing something else, respectively. Beginning in 2011, those who report providing eldercare in the last three months are asked comparable questions about secondary eldercare. Not all primary activities are eligible for concurrent secondary activities. For example, a person may not report providing secondary childcare when their primary activity is coded directly as sleep or childcare; however, they may report providing both eldercare and childcare simultaneously (e.g., reporting their primary activity as caring for a child and noting that they were also providing secondary eldercare during the activity, or reporting the provision of both secondary childcare and eldercare at the same time).

**Who Records** report who else was with the respondent (or in the room with them) during the activity. If household members or non-resident children of the respondent are present during the activity, the time diary reports details about which persons are present. If non-household members (other than non-resident children) are present, the type of person or people they were with is recorded, but not the specific individual(s) or the number of individuals in each relationship category. For example, an adolescent in the ATUS may identify the specific coresident parent present for activities; it is therefore feasible to compare the types and duration of activities adolescents might spend with one parent versus another parent. However, the ATUS does not record the total number of friends present during an activity or differentiate among them, meaning it is not possible to know if a respondent spent an afternoon with one or more friends, or met a new friend for each activity. Beginning in 2010, respondents report on the presence of others while working and can differentiate between those who might be present during paid work activities at a work setting such as boss or manager, supervisees, coworkers, or customers. Because respondents can report being with several people during an activity, a single activity record can have many “who” records associated with it.

**Person Records** include demographic information about the respondent, both that collected at the time of the ATUS interview (e.g., employment, work hours, age) and information gathered at the time of the final CPS interview that occurred two to five months before the ATUS interview (e.g., education, race). Activity Records, and the Who Records associated with them, are organized by person. While there is only one respondent with a time diary per household, demographic information about all household members is also included in the file. As with the respondent’s characteristics, some of this is updated at the time of the ATUS interview, though most of the reported characteristics come from the CPS. Information about each specific household member is stored on a person-specific record such that information about the ATUS respondent’s spouse or youngest child, for example, is stored on the records that represent those individuals. Files organized this way avoid the proliferation of variables that would be necessary if, for example, all of the information on five household members was repeated on the ATUS respondent’s record.

**Household Records** are employed in IPUMS ATUS for prudence. All Person Records in the data—ATUS respondents, their household members, and non-resident children—are organized into households. Rather than repeating, for example, geographic information or number of household members on each person’s record, we simply store it on a single Household Record.

**Eldercare Records** are generated for ATUS respondents who provide eldercare; these respondents share details about the recipients of the care that they provide. IPUMS ATUS then creates a record in the data file for each care recipient, describing the relationship of the recipient to the ATUS respondent, their age, how long the ATUS respondent has been providing care to them, and, if the recipient is coresident, a line number enabling linkage of the record to the person’s full record in the data file. These records are also stored separately from the main set of information on ATUS respondents.

### 2.2. Challenges of the ATUS

Taking full advantage of the wealth of information contained in the ATUS data requires advanced data management and manipulation skills. Analyzing specific activities, activity sequences, and/or population subgroups often requires pooling together multiple years of data. Each year of data includes up to ten files that must be merged to utilize the full detail. The BLS offers “combined” files that contain all years of ATUS data for specific file types, but the separate file types still need to be collated prior to analysis.

Merging ATUS files together requires a clear understanding of the inter-relationships between files (see Table 1) and record types for different levels of analysis (e.g., respondents, activities, household members). Once the files have been appropriately collated, analysts must still summarize and transpose information across record types to produce estimates that are meaningful and feasible to interpret. For example, estimating differences in parents’ time spent socializing with friends by caregiver status requires combining information across five different BLS data files: respondent (respondent demographics), activity (socializing), who (presence of friends), eldercare (caregiver status), and replicate weights (for producing standard errors that account for the complex sample design of the ATUS).

ATUS data are greatly enhanced through linkage with the CPS, from which the ATUS sample is drawn. The annual ATUS data releases include an ATUS-CPS data file that attaches variables collected during the final CPS interview onto the ATUS respondent and members of their household. However, to utilize the longitudinal linkage and full range of supplemental data available in the CPS, users must still link individual records between the ATUS and the CPS using linking keys that vary across time.

## 3. Methods

IPUMS ATUS offers tools that increase the usability of the ATUS by harmonizing data, providing integrated and high-quality documentation, and streamlining data management and manipulation tasks. These innovations make it easier for analysts to wrangle the data to explore the countless research questions that the ATUS data are suitable for answering. The comparative advantage of IPUMS ATUS is demonstrated in our usage statistics: over 10,000 researchers have registered accounts to use IPUMS ATUS data and have collectively created more than 43,500 customized datasets with IPUMS ATUS.

### 3.1. Harmonization

To use data across multiple years, researchers must first reconcile differences in variable names and codes to create measures that are comparable across their time period of interest. It is inefficient for each individual researcher using multiple years of data to review all of the relevant PDF-formatted documents required to create variable coding schemes that facilitate interoperability, particularly for data collections like the ATUS that span decades. Writing code to harmonize these variables is error prone. Furthermore, each individual researcher would likely take a slightly different approach, making their results difficult to replicate.

For over thirty years, IPUMS has been providing researchers with access to a common starting point for combining data across time and space. We generate harmonization crosswalks, which we call “translation tables”, that are readable by both humans and machines. The crosswalks employ structured metadata that describe how to translate year-specific codes into a coding scheme that is consistent (or “harmonized”) across time.

Table 4 presents a simplified version of the translation table for the IPUMS ATUS variable ENOUGHFD, which is part of the Eating and Health module and reports if there was enough food to eat in the household in the last 30 days. The original codes from 2014 to 2016 and 2022 to 2023, shown on the right, are recoded into the harmonized codes in the left-most column. IPUMS ATUS employs a composite coding scheme for this variable. In contrast to the 2014–2016 codes, the 2022–2023 response options included additional detail for the “Enough to eat” category, differentiating between whether or not the foods are the kinds that the respondent/household members want to eat. For a comparable time series, researchers may use only the first digit of the ENOUGHFD variable, thereby collapsing the detailed categories present in 2022–2023 into the more generic code for “Enough to eat” that is available in 2014–2016. Researchers working with only 2022–2023 data may use the two-digit version of the codes that contain additional detail.

Each translation table is accompanied by other structured metadata. The additional metadata provide a brief description of the variable, the universe, comparability issues of note, variable availability across time, associated data quality flags, and the original questionnaire text associated with this variable in each year.

### 3.2. Data Access System

These harmonized data and integrated documentation are delivered to users via the IPUMS web-based data access system. The interactive IPUMS data access system allows users to create customized datasets that combine multiple years of data, but only include the variables they need. The same structured metadata that dictate how to recode the original data into a harmonized version also populate the variable-level documentation on the IPUMS websites. This ensures that the data and documentation move through our system in lockstep.

The IPUMS ATUS data access system draws on harmonized, multilevel, hierarchical data files (as described in Table 1) that combine information from the many different data files released for the ATUS each year. With the data access system, users can easily combine multiple years of data, and they can choose data structures appropriate for their analysis. Users can choose between data that are rectangularized on persons (appending household-level characteristics onto the person) or the activity (appending person-level characteristics onto activities), or request a hierarchical file that organizes different records by their inter-relationships (e.g., who records nested within activities, which are nested within persons who are organized into households). The amount of data manipulation required by the user depends on what they want to do with the data. Rectangular files typically require the least manipulation. Multilevel hierarchical files, on the other hand, offer the most flexibility but require more data management.

After defining the contents of their customized dataset, users select the format of their data file. By default, IPUMS ATUS provides a fixed-width (.dat) file and support for reading the file into Stata, R, SPSS, SAS, and Python. Alternatively, users can request a file formatted as a CSV or the appropriate native format to be opened directly in Stata, SPSS, or SAS.

Every customized IPUMS ATUS dataset request is logged in a user’s account. The criteria for each customized dataset are preserved and can be resubmitted to regenerate the dataset or used as a starting point for creating a new customized dataset. IPUMS delivers archival standard Data Documentation Initiative (DDI) codebooks along with every customized dataset. The codebook documents the digital object identifier (DOI) and version of the data used to create that specific customized dataset, as well as the metadata for the variables and samples the dataset includes.

Beyond providing a direct way to access harmonized data, generating and downloading a customized dataset from IPUMS ATUS is much more efficient than working with the original versions of the data. The average time it takes for IPUMS ATUS to generate a customized dataset is under five minutes. This is substantially less time than it would take users to download the original ATUS data files, manually collate the files, and then combine multiple years of data, much less harmonize the variables across time.

### 3.3. Custom Time Use Variables

Creating variables that summarize daily time use from a respondent’s time diary requires advanced data manipulation. In recognition of the difficulty to create these summary measures, the annual BLS release of the ATUS includes a file that reports the total time respondents spent in each six-digit activity code (e.g., laundry, working at main job). However, many users will still need to modify these variables, combining similar categories together (e.g., there are 33 unique activity codes for attending sporting events [6]) or refining measures of time spent in an activity based on other details in the time diary (i.e., location, secondary activity, time of day, co-presence). The IPUMS ATUS Time Use Variable (TUV) builder provides a graphical user interface that allows researchers to easily define their own TUVs, either modifying pre-existing TUVs or creating a variable from scratch. For example, the TUV builder makes it easy to create a variable that reports time spent doing housework while also providing childcare. A user can modify the pre-existing TUV that reports the time spent in the primary activity “Household activities: Housework” (BLS_HACT_HWORK) to only count time when the person also reports providing secondary childcare. The IPUMS ATUS data access system leverages our structured metadata and information from the user’s custom TUV definition to execute the required complex programming to create the new housework and secondary childcare TUV and include it in the user’s customized dataset.

Custom TUVs from IPUMS allow users to leverage the full array of information in the ATUS to examine activities under highly specific circumstances. The feature allows users to stipulate whether or not to include activities based on timing, location, presence of others, and any co-occurring secondary activities. These custom TUVs are saved and stored in a user’s IPUMS ATUS account so they can be reused easily in future customized datasets. As of this writing, IPUMS ATUS users have created 34,500 unique custom TUVs and have included pre-existing TUVs in more than 20,000 of their customized data files.

Providing users with a point-and-click interface to generate these complex measures of time use not only increases the accessibility of these complex data to a broad range of researchers but also saves researchers extensive time manipulating data to generate such summary measures. The specific criteria used to create a TUV are easy to identify and share.

### 3.4. Linking to CPS

In addition to the richness and flexibility of the ATUS, another core strength of the data is that they can be linked to the Current Population Survey (CPS). The CPS is a monthly household survey with a panel component. Households selected to participate in the CPS are interviewed eight times over 16 months following the “4–8-4 rotation pattern”, where households are interviewed for four consecutive months after entering the CPS, take an eight-month break, and then are interviewed for four more consecutive months before exiting the survey. Individuals and households can be linked across these eight interviews. A new rotation group enters the CPS panel each month; there are eight distinct rotation groups actively responding to the CPS in any given month, each at a different point in the rotation pattern.

Each month, CPS household members respond to a series of demographic and labor force questions. Many months include topical supplements in addition to the “basic monthly” data collection; these supplements are generally collected on a regular (annual, biannual) basis and cover topics such as displaced workers, job tenure, child support, disability, fertility, volunteering, education, tobacco use, civic engagement, and food security. The Annual Social and Economic Supplement (ASEC), the most widely known and used of the CPS supplements, collects detailed information about income and program participation as well as health insurance coverage and access.

Households that have completed their eighth CPS interview make up the sampling frame for the ATUS. Because of the CPS panel component, the ATUS can be linked to both the monthly CPS and to CPS supplements from any point in the panel. Linking the ATUS to a single month of the CPS or the full CPS panel further enriches the data, but it is challenging. First, there are no singular linking keys in the original data. Users must concatenate multiple variables to uniquely identify households and individuals, and these variables change across time. There are a variety of related issues with CPS linking that we describe in depth elsewhere [8–11]. Second, there is a lag of varying duration between the eighth CPS interview and the ATUS interview. Because the ATUS is fielded continuously, each annual ATUS file can be linked to up to fifteen months of monthly CPS data, requiring substantial file management for even a single year of ATUS data. Third, taking advantage of the full CPS panel when linking the ATUS to the CPS requires that data users also link across CPS interviews or supplements.

Working with IPUMS ATUS reduces the challenges of linking ATUS and CPS data because IPUMS also disseminates a harmonized version of the CPS. IPUMS researchers created a variable that uniquely identifies and links individuals across the CPS [8–13]. We include this linking key in IPUMS ATUS as well, facilitating efficient linkages with the CPS. Users can simply include the linking key in their customized IPUMS ATUS and IPUMS CPS data files and merge their two custom data files. Like IPUMS ATUS, IPUMS CPS makes it easy to pool multiple months of CPS data into a single file. Rather than downloading CPS monthly and supplement data for each of the 15 months that are eligible to link to one year of ATUS data, users can request a single data file from IPUMS CPS that will include all months and supplements that link to their focal year(s) of the ATUS.

### 3.5. Reproducibility

IPUMS ATUS facilitates reproducibility by providing the research community with a common starting point for working with harmonized time diary data and the ability to create well-documented, customized time use variables. In addition to these features that promote reproducibility more broadly, we assign a DOI to the IPUMS ATUS database. The DOI and version number of the database are updated annually with the release of the latest year of ATUS data. With each version update, we also preserve a snapshot of the full IPUMS ATUS database in case researchers need to access a previous version of the database. Changes between versions (i.e., revisions to coding schemes, corrections) are documented in our public-facing revision history [14].

## 4. User Notes

### 4.1. Applications of IPUMS ATUS Data

The diverse applications of IPUMS ATUS data demonstrate the flexibility and relevance of the data to a wide variety of topics. The data are used to capture key aspects of daily life and differences by sociodemographic characteristics [15–18] and are a vital source of information on caregiving for children [19–21]. The Eldercare data contextualize adult caregiving alongside caregiver time use and resources [22,23]. The Well-being module has been used extensively to assess the well-being of couples [24–26], parents [27,28], and many other groups and contexts [29–31]. The Eating and Health module has been used to study associations between time spent in food-related activities and health and economic outcomes [32–34], and the Leave and Job Flexibility module enables research on the impact of paid and unpaid leave, including on topics ranging from parenting [35] to schedule control [36].

### 4.2. Considerations for ATUS Data

IPUMS ATUS effectively addresses the major complexities of working with time diary data from the ATUS. There are, however, considerations that data users should bear in mind when using these data.

First, weights must be used when computing estimates with the ATUS. Interpreting unweighted tabulations will produce misleading results. Weights are calculated and provided by the BLS with the original ATUS data. The provided weights account for the oversampling of certain demographic groups to ensure adequate sample size of those groups, the oversampling of weekend days, and differential response rates by demographic group and day of the week. In addition to weights for the time diary data, there are separate weights for modules.

Second, the recommended weights for most analyses with IPUMS ATUS are appropriate for producing annual estimates. The weights sum to the number of person-days in the country in a quarter. Using ATUS data to produce person-level analyses that do not include time use variables requires dividing the weight variable by the number of person-days in the country in the quarter.

Third, the ATUS can be used to generate population-level estimates, including estimates for demographic subgroups and specific geographic areas. While the ATUS captures only one diary day per respondent and it is possible that the day was atypical for them, aggregations of the data are representative of the American population. Ensuring that the unweighted sample size is sufficiently large before weighting analyses is pivotal for generating representative statistics.

Fourth, data users need to also consider how to handle zeroes in their time use variables, which indicate that activities were not observed on the diary day. Zeroes can result for one of two reasons—because the respondent did not do the activity on the diary day and because they never do the activity—which cannot always be differentiated. For example, one would expect the parent of an infant to spend time caring for the child on the diary day. Sometimes parents report no childcare time. More than likely, no time in childcare reflects a day when the parent was away from home for an extended workday rather than that they never care for the child. Exercising is a more ambiguous case. Some people never exercise, some exercise daily, and still others have an exercise routine that is regular but not daily. Except in the case of the Eating and Health module from 2014 forward, there is no variable to help differentiate between these types of people. We simply know whether they exercised or not on the ATUS diary day. Handling of zeroes, then, requires that users clearly document and justify their approach, and discuss the implications for their results. Depending on the application and interpretation, it may be appropriate to retain these zeroes or to restrict analyses only to those who performed a focal activity [37,38].

Finally, data users should note that while the ability to link to the CPS is a strength of the data, the respondent’s circumstances may have changed between their CPS interview(s) and the ATUS. A limited number of demographic measures are collected or updated at the time of the ATUS; most come from the eighth CPS interview. For research focused on certain types of transitions (e.g., life course transitions such as graduation, parenthood, or retirement) or population groups that might experience rapid change (e.g., low-wage workers) it may be inappropriate to use information gathered at the time of the CPS to contextualize time use reported in the ATUS. IPUMS ATUS denotes variables that report data collected at the time of the eighth CPS interview by appending the suffix “*_CPS8” to the variable name to increase the transparency about the source of the data.

### 4.3. The Future of IPUMS ATUS

IPUMS ATUS plans to continue enhancing the time diary from the ATUS. We will add new variables that are not in the original ATUS data but are of great value to researchers: harmonized variables that account for regular updates to the occupation and industry classification schemes; sleep quality variables; and geography variables that leverage the complex spatial relationships among metropolitan areas, cities, and counties to derive additional detail from the provided geographic identifiers [39]. We are also developing additional resources for linking ATUS and CPS data, including a tool that allows users to visualize which months and supplements of the CPS an ATUS respondent can link to.

IPUMS ATUS is the most widely used of the three time diary resources that IPUMS offers. IPUMS AHTUS and IPUMS MTUS harmonize historical time diary data and international time diary data, respectively. All ATUS samples are currently available in the IPUMS Multinational Time Use Study (MTUS), where the data can be used for cross-national comparisons. Soon, IPUMS will also integrate all years of the ATUS into the IPUMS American Heritage Time Use Study (AHTUS) to facilitate use of the continuously fielded ATUS data with a series of stand-alone time diary datasets going back to 1930.

### 4.4. Conclusions

Time diary data offer a unique and valuable combination of structure and flexibility that allows quantitative researchers to explore many facets of human behavior. The American Time Use Survey—the first federally administered, ongoing survey on time use in the United States—is an extraordinary and unparallelled resource for understanding how Americans spend their time. Although the ATUS provides myriad possibilities for social science and health research, the complex hierarchical record structure makes the data difficult to use. IPUMS ATUS provides a version of the ATUS data that has been harmonized across time and is available through an online platform that streamlines data analysis by simplifying both data file management and the creation of customized time use variables. Thanks to the generous support of the Eunice Kennedy Shriver National Institute of Child Health and Human Development, IPUMS ATUS data and enhancements are available to the research community free of charge and we continue to identify additional opportunities to increase the accessibility of this valuable resource.

## Figures and Tables

**Table 1.** Hierarchical data structure.

Record Type	Description	BLS Data File(s) Represented
H	Household—one per ATUS respondent	Respondent, ATUS-CPS
P	Person—one per ATUS respondent and for each household member	Respondent, Roster, ATUS-CPS, Case History, Weights
A	Activity—Many per ATUS respondent	Activity
W	Who—One or more per activity	Who
R	Eldercare Recipient—One or more per ATUS respondent who provides eldercare	Eldercare Roster

**Table 2.** Activity coding scheme for household activities.

Full Activity Code	Major	Intermediate	Detailed *	Description
020000	02	n/a	n/a	Household activities
020100	02	01	n/a	Housework
020101	02	01	01	Interior cleaning
020102	02	01	02	Laundry
020103	02	01	03	Sewing, repairing, and maintaining textiles
020104	02	01	04	Storing interior household items, including food
020199	02	01	99	Housework, n.e.c.
020200	02	02	n/a	Food and Drink Preparation, Presentation, and Clean-up
020300	02	03	n/a	Interior Maintenance, Repair, and Decoration
020400	02	04	n/a	Exterior Maintenance, Repair, and Decoration
020500	02	05	n/a	Lawn, Garden, and Houseplants
020600	02	06	n/a	Animals and Pets
020700	02	07	n/a	Vehicles
020800	02	08	n/a	Appliances, Tools, and Toys
020900	02	09	n/a	Household Management
029900	02	99	n/a	Household Activities, n.e.c.

* For brevity, only the six-digit detailed activity codes nesting within the intermediate activity “Housework” are listed.

**Table 3.** Sample time diary data.

Start Time	Stop Time	Activity	Location	Secondary Activity	With Whom *
4:00:00	6:00:00	Sleeping	n/a^†^	n/a	n/a
6:00:00	6:15:00	Washing, dressing, and grooming oneself	n/a	n/a	n/a
6:15:00	6:20:00	Food and drink preparation	Home	n/a	Alone
6:20:00	6:35:00	Eating and drinking	Home	n/a	Spouse, Child1, Child2
6:35:00	6:50:00	Travel related to work	Car	n/a	Alone
6:50:00	6:55:00	Waiting for the train	Other place	n/a	Alone
6:55:00	7:20:00	Travel related to work	Train	n/a	Alone
7:20:00	9:30:00	Work, main job	Work	n/a	Alone
9:30:00	10:30:00	Socializing, relaxing, and leisure as part of job	Work	n/a	Coworkers
10:30:00	10:35:00	Washing, dressing, and grooming oneself	n/a	n/a	n/a
10:35:00	11:50:00	Work, main job	Work	n/a	Alone
11:50:00	11:55:00	Travel related to eating and drinking	Walking	n/a	Coworkers

* For illustrative purposes, we show “who” data alongside activities, though they are not available natively in this format.

† Location information is not available for a subset of activities (e.g., personal activities, sleeping).

**Table 4.** Simplified translation table for IPUMS ATUS ENOUGHFD variable.

Harmonized Codes		Original Codes	
Composite code	Label	2014–2016	2022–2023
10	Enough to eat	1	*
11	Enough of the kinds of food I/we want to eat	*	01
12	Enough, but not always the kinds of food I/we want to eat	*	02
20	Sometimes not enough to eat	2	03
30	Often not enough to eat	3	04
96	Refused	−3	−3
97	Do not Know	−2	−2
98	Blank	−1	−1
99	NIU (Not in universe)	blank	blank

* There are no codes in the original data that correspond to the harmonized codes in the left-most column.

## Data Availability

IPUMS ATUS data and documentation are freely available to registered users. Interested users can create a free account to access IPUMS ATUS at atus.ipums.org.

## Footnotes

1 There are two BLS files that IPUMS ATUS does not integrate. There is extremely limited demand for the “Call history” file. In lieu of releasing the “Activity Summary” file, which provides a limited number of time use variables, IPUMS ATUS offers a tool to create custom time use variables (see Custom Time Use Variables section).

## Abbreviations

The following abbreviations are used in this manuscript:

AHTUS: American Heritage Time Use Series
ASEC: Annual Social and Economic Supplement
ATUS: American Time Use Survey
BLS: Bureau of Labor Statistics
CPS: Current Population Survey
CSV: Comma-Separated Values
DDI: Data Documentation Initiative
DOI: Digital Object Identifier
MTUS: Multinational Time Use Series
PDF: Portable Document Format
TUV: Time Use Variable
U.S.: United States
